# Root Canal Dentin Microhardness after Contact with Antibiotic Medications: An In Vitro Study

**DOI:** 10.3390/dj12070201

**Published:** 2024-06-29

**Authors:** Amanda Palmeira Arruda Nogueira, Renata Grazziotin-Soares, Adriana Marques Mesquita Leal, Sérgio Alves Guida Freitas Júnior, Bruna Laís Lins Gonçalves, José Bauer, Meire Coelho Ferreira, Ceci Nunes Carvalho

**Affiliations:** 1 School of Dentistry, CEUMA University, São Luís 65075-120, MA, Brazil; nogueira.amanda@discente.ufma.br (A.P.A.N.); mmladriana@hotmail.com (A.M.M.L.); sergio2477146@ceuma.com.br (S.A.G.F.J.); brunalais25@hotmail.com (B.L.L.G.); meire.ferreira@ceuma.br (M.C.F.); 2 Department of Oral Biological Medical Sciences, Faculty of Dentistry, University of British Columbia UBC, Vancouver, BC V6T 1Z4, Canada; renata.grazziotin@ubc.ca; 3 Dental Materials Laboratory, School of Dentistry, University Federal of Maranhão (UFMA), São Luís 5085-582, MA, Brazil; jose.bauer@ufma.br

**Keywords:** dental pulp, dentin, hardness test, regenerative endodontics, root canal medicament, dentistry, endodontics

## Abstract

**Background:** Antibiotic pastes used as intracanal medication in cases of revascularization therapy might cause negative effects on tooth properties, such as a reduction in dentin microhardness. This in vitro study investigated dentin microhardness in three different locations distancing from the canal lumen after 20 days of treatment with a tri-antibiotic paste (ciprofloxacin, metronidazole, and minocycline), and with a double-antibiotic paste (ciprofloxacin and metronidazole), with calcium hydroxide [Ca(OH)_2_] Ultracal^TM^ XS-treated dentin as comparison. **Material and Methods:** Human mandibular premolars (n = 48) had the root canals cleaned and shaped and were used to produce dentin slices. Dentin slices remained immersed in the medications for 20 days. The Knoop microhardness (KHN) test was performed before (baseline/Day-0) and after treatment (Day-20) with the medications. Indentations were made at 25 µm, 50 µm, and 100 µm distances from the root canal lumen. The KHN was compared intra-group using Wilcoxon’s test. Independent groups were compared using Mann–Whitney’s and Kruskal–Wallis’ tests, at α = 5%. **Results:** The microhardness in all the tested groups was reduced at Day-20 in comparison with Day-0 (*p* < 0.001) (intra-group comparison/same distances). The Day-0 values were similar, and the Day-20 values were higher for the Ca(OH)_2_ group (*p* < 0.05) (comparison between groups/same distances). **Conclusions:** Calcium hydroxide for 20 days would be preferred rather than antibiotic pastes to minimize the expected reduction in dentin microhardness during regenerative procedures.

## 1. Introduction

The maintenance of teeth within the oral cavity and their correct functioning are seen as critically important, and advanced dental procedures in the field of Endodontics fall within them [1]. The great challenge of endodontic treatment is not to combat the bacteria present in the root canal, whether through mechanical removal—the use of endodontic files—or chemical removal—the use of irrigating substances [2]. These micro-organisms are capable of inducing pulp inflammation, often leading to pulp death [2], and, sometimes even with antisepsis obtained through these techniques, the preparation is considered temporary and partial, and, because of this, the use of intracanal medications ends up being necessary [1,2,3,4].

Intracanal medication is used in the tooth root canal between dental appointments. During that period, the medication continually releases active substances—which give the medication its antimicrobial potential, sealing ability, and biocompatibility [5,6,7]. Several intracanal medications can be used, such as corticosteroids (Otosporin^®^), triple antibiotic paste (PTA), double antibiotic paste (PDA), 2% chlorhexidine (CXH), and calcium hydroxide [Ca(OH)_2_] [2], with the treatment being the indicator of the best medication to be used, such as pulp revascularization therapy [2,8,9].

Although they have several advantages, such as the antimicrobial and anti-inflammatory action mentioned above, some intracanal medications may negatively alter the tooth dentin re-resistance—a drawback for the endodontic/root canal therapy [10]. Regenerative Endodontic Treatment, also known as Pulp Revascularization, is performed on permanent teeth diagnosed with pulp necrosis and incomplete rhizogenesis, as an alternative therapy to apexification [11]. This treatment is a major challenge within Endodontics, since, when there is a necrotic process, the elimination of bacteria through filing is essential, but, in this case of an open apex, the use of files ends up further reducing the thickness of the walls, affectingexisting dentines. Therefore, the use of intracanal medications is the way to eliminate these micro-organisms [11,12].

The objective of Pulp Revascularization is the creation of new pulp tissue from apical papilla stem cells (DPSCs) and other cells present in the peri-apical tissues induced by apical bleeding [12]. Because of this objective, the intracanal medication used needs to be bio-compatible with the tissues and help induce the migration of DPSC into the root canal. Currently, the most used medications are calcium hydroxide, double-antibiotic, and trian-tibiotic paste [11,12].

Dentin microhardness is an important property for teeth undergoing revascularization therapy. Revascularization therapy is indicated for immature permanent teeth. Those teeth usually have thin root canal walls and an open apex before the therapy [8,9]. Following the therapy protocol, a thicker intracanal structure is then expected [13,14]—which should strengthen the tooth structure, allowing the tooth to remain in function [15,16]. Although the formation of intracanal hard tissue may be favorable for tooth mechanical resistance, weak intracanal hard tissue could be formed—because of the reduced dentin microhardness after a period of contact with antibiotic medications. Ultimately, this weak formed structure would contribute to potential tooth fracture [13,14,15,16].

Scientific evidence shows that regenerative procedures are effective, with high percentages of success (78% to 100%) [17,18,19,20]. However, despite the great success, some authors have stated that regenerative procedures might fail [20,21,22,23,24,25,26]. The reasons for failure are root re-sorption, persistent infection, and tooth fracture. Tooth fracture is linked to a longer duration of intracanal medication, which may induce negative modifications in the tooth properties, such as changes in the mineral content ratio, increased permeability and solubility, and reduced microhardness of the root canal dentin [27,28].

Studies found a significant reduction in microhardness of root dentin treated with antibiotic pastes when compared to untreated root dentin [29,30,31,32]. However, some studies did not use the values of calcium-hydroxide-treated dentin as a comparison to antibiotic-treated dentin [33,34], and it is accepted that calcium hydroxide is also capable of reducing dentin microhardness [25,35,36,37]. This point is a gap within the literature, justifying, therefore, the present study.

Considering the importance of having detailed knowledge about the potential negative effects that antibiotic pastes might cause on the root dentin during regenerative procedures, the purpose of this study was to investigate dentin microhardness in three different locations distancing from the canal lumen after 20 days of treatment with a tri-antibiotic paste (TAP) (ciprofloxacin, metronidazole, and minocycline), and with a double-antibiotic paste (DAP) (ciprofloxacin and metronidazole), with calcium hydroxide-treated dentin as comparison. The expectation was that antibiotic pastes would reduce the dentin microhardness to a lesser extent than calcium hydroxide. The null hypothesis was that there would be no difference in dentin microhardness treated with TAP, DAP, or calcium hydroxide paste [Ca(OH)_2_].

## 2. Materials and Methods

### 2.1. Study Design and Ethics

This is an in vitro experiment that used human teeth to measure dentin microhard-ness depending on which intracanal medication was used: (1) tri-antibiotic paste com-posed of ciprofloxacin, metronidazole, and minocycline (TAP); (2) double-antibiotic paste composed of ciprofloxacin and metronidazole (DAP); and (3) calcium hydroxide paste, (Ul-tracalTM XS, Ultradent, South Jordan, UT, USA) [Ca(OH)_2_]. The project protocol was approved by the Ethics Committee Board where the experiment was conducted (approval number #CAAE 44282315.5.0000.5084).

### 2.2. Sample Size Calculation and Teeth Collection

Sample size calculation was performed to compare the means of microhardness (KHN) between groups. The following parameters were adopted: confidence level of 95%, power of 80%, standard deviation of 5, and a minimum difference to be detected between the groups of 5 points in the mean microhardness. The n obtained was 16 samples/specimens per group. As each tooth would originate 4 specimens after sectioning (more information is shown below in Section 2.3), there was a need for 48 teeth. The sample size calculation and the methodology followed a previous study [33].

With forty-eight unidentifiable and untraceable human mandibular premolars, the extracted teeth were all caries-free. All the teeth were extracted for orthodontic, periodontal, or other dental treatment reasons. Organic material was removed from the root surface with curettes. Teeth were stored in 0.1% thymol at 4 °C and used within 6 months after extraction. Radiographs and visual assessments under magnification were used for teeth selection. Teeth were included if they had a single canal, completed closed apex, and no signs of calcification, internal resorption, or previous endodontic treatment. We exclude the teeth that had large cavities, fracture roots, and extensive restorations.

### 2.3. Specimen Preparation (Root Slice Fabrication and Treatment with Medication Pastes)

Teeth were decoronated at the cementoenamel junction using a cutting machine (Isomet 1000 Precision Saw Buehler Ltd., Lake Bluff, IL, USA). Working length for each root was measured by inserting a size #10 K-file (Maillefer, Dentsply Industria e Comercio Ltda, Petropolis, RJ, Brazil) into the root canal until the tip of the file was visualized in the apical foramen. The file was then pulled back 1 mm—under a stereoscopic magnifying glass of 25× (Baush, Lomb, Rochester, NY, USA).

Root canals were negotiated with a size #10 K-file, and then cleaned and shaped with a single reciprocating nickel–titanium instrument (Reciproc R50—VDW, München, Germany), an instrument that has a taper of 0.06, under irrigation with 10 mL of 1% sodium hypochlorite at pH 11. EDTA was not used. The Reciproc R40 file was used in X-Smart Plus (Dentsply, Maillefer, Switzerland) programmed in Reciproc All mode. During instrumentation, the root canals were irrigated with 2 mL 1% NaOCl (Fórmula e Ação, São Paulo, SP, Brazil) and, finally, rinsed with sterile saline to remove dentin debris.

Four root specimens/slices (2 mm thickness) were obtained from the middle third of each root using the cutting machine. The specimens were embedded in PVC tubes with acrylic resin (TDV, Pomerode, SC, Brazil) with one of the dentin surfaces (and canal space) facing up, and free of resin. The dentin surfaces were sanded with 400#, 600#, and 1200# grids of sandpaper, and then polished with felt cloths soaked in paste diamond (Diamond, FGM, Joinville, SC, Brazil) at low-speed rotation [38]. After cleaning the specimens in an ultrasonic vat under distilled water for 3 min and drying them with paper towel, specimens were divided at random in 3 groups (n = 16) according to the intracanal medication to be used.

The antibiotic pastes were fabricated by grounding one tablet of each antibiotic (500 mg) to obtain a mixture 1:1:1 (TAP) or 1:1 (DAP) by weight, measured in a precision scale [34,35]. The pastes were then produced by mixing the powder with saline solution. The following powder/liquid ratios were used: (1) TAP = 3:1, and (2) DAP = 2.5:1. Antibiotic pastes were applied over the dentin samples and into individual containers using a metallic spatula. UltracalTM XS (Ultradent, Itaici—Indaiatuba, São Paulo, Brazil) is a premixed Ca(OH)_2_ paste for direct application, and it was applied over the samples and into the containers using 29 ga NaviTip tips with single sideport (Indaiatuba, SP, Brazil).

The specimens (root discs) were placed in Petri dishes, and then assigned randomly to the three treatment groups. The medicaments were placed in the Petri dishes, and the discs were completely covered with the mixture. The Petri dishes were covered with stretch film and remained in an incubator at 37 °C and 100% humidity (distilled water) for 20 days. After treatment, specimens were cleaned in ultrasonic vat for 3 min and dried.

### 2.4. Microhardness Test (Before and After Treatment with Medications)

Dentin microhardness were acquired at Day-0 (baseline/before medication treatment) and after 20 days immersed in the medication (Day-20). Microhardness was measured with a Knoop indenter at 40× magnification (Shimadzu HMV-2000; Shimadzu Corporation, Kyoto, Japan) A pyramid-shaped diamond indenter was used to analyze them with a load of 10 g for 15 s. The mean length of the two diagonals in each indentation was used to calculate the Knoop microhardness value [KHN]. The Knoop method is often used when lighter loads are needed (as in our study), where very small loads are recommended to be able to note changes in human dentin microhardness—and the shape of the probe is suitable for samples needing indentations close together or on the edge. Because of the large difference between the long and short Knoop diagonals, the Knoop indenter is often better suited for determining variations of hardness over very small distances compared to the Vickers indenter. It can also offer higher levels of precision than the Vickers method, due to the fact that the longitudinal diagonal of the indenter is higher [33].

**Figure 1:**
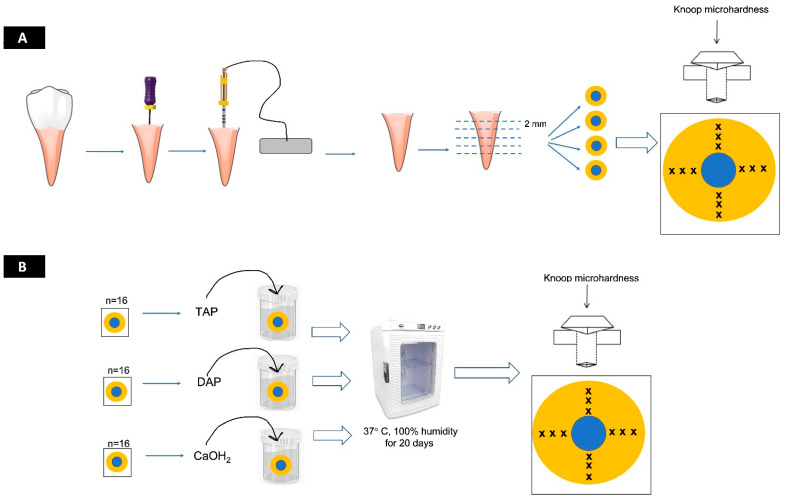
Graphical design showing the summary of the study methodology: (**A**) Root slice fabrication and microhardness test at baseline/Day-0. Forty-eight human extracted premolars were used to produce slices. Slices were then submitted to Knoop microhardness test (12 indentations in each sample at 25, 50, and 100 µm). (**B**) Treatment of dentin with medication pastes and microhardness test at Day-20. Dentin slices remained immersed in the medications for 20 days and were submitted to Knoop microhardness test. TAP = tri-antibiotic paste composed of ciprofloxacin, metronidazole, and minocycline. DAP = double-antibiotic paste composed of ciprofloxacin and metronidazole. Ca(OH)_2_ = calcium hydroxide paste (Ultracal^TM^ XS, Ultradent, South Jordan, UT, USA).

**Figure 2:**
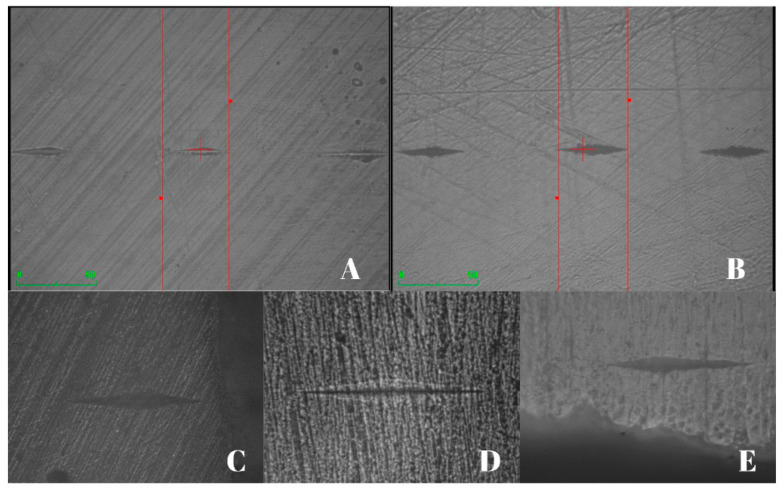
Dentin microhardness test. (**A**,**B**): indentations on the dentin surface with a magnification of 50 µm. (**C**–**E**): indentations on the dentin surface with a magnification greater than 50 µm. The “+” in red shows the center of the sample.

Twelve indentations were performed for each specimen: 3 indentations in the upper region (at 25 µm, 50 µm, and 100 µm from the root canal lumen), 3 indentations in the lower region (at 25 µm, 50 µm, and 100 µm), 3 indentations in the right region (at 25 µm, 50 µm, and 100 µm) and 3 indentations in the left region (at 25 µm, 50 µm, and 100 µm). Each disc received a series of three indentations at points around the pulp space 1 mm from the canal wall. The representative microhardness value for each distance in the specimen was obtained by averaging the result of the 4 indentations (upper, lower, left, and right) performed in the distance (Figure 1 and Figure 2). All tests carried out were guided by the ASTM E384-22 standard [39].

### 2.5. Data Analysis

Day-0 and Day-20 dentin microhardness values (KHN) were compared intra-group using Wilcoxon’s test. Dentin microhardness for independent groups [TAP, DAP, and Ca(OH)_2_] in each distance from the canal lumen (25 µm, 50 µm, and 100 µm) were compared using Mann–Whitney’s and Kruskal–Wallis’ tests. Analyses were performed using the Statistical Package for Social Sciences (SPSS for Windows, version 21.0, SPSS Inc. Chicago, IL, USA), at α = 5%.

## 3. Results

**Figure 3:**
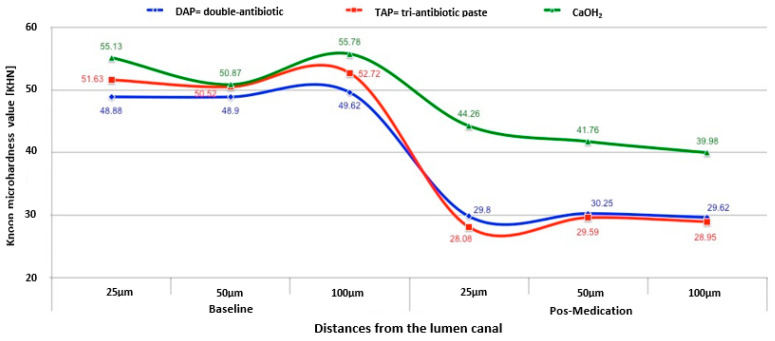
Baseline and post-medication dentin microhardness (HKN) mean values, at different distances from the root canal lumen.

There was no statistical difference for the baseline (Day-0) microhardness considering the specimens allocated for the three groups (*p* > 0.05), demonstrating the effectiveness of the random allocation. There was no statistical difference for the baseline microhardness considering the distance from the canal lumen (25 µm, 50 µm, and 100 µm) (*p* > 0.05) (Figure 3).

In the intra-group comparison (same distances), the microhardness values (KHN) in all the tested groups [TAP, DAP, and Ca(OH)_2_] were significantly reduced on Day-20 of the experimental time in comparison with Day-0 (*p* < 0.001 for the three medications). Individual results for each experimental time are shown in Table 1 and Table 2).

In the comparison between groups (same distances), the Day-0 measurements, regardless of the measured distance, were similar to each other (*p* > 0.05). For the Day-20 measurements, the Ca(OH)_2_ group had higher microhardness values than TAP and DAP, regardless of the measured distance (*p* < 0.05). The TAP and DAP groups had similar values, regardless of the measured distance (*p* > 0.05).

## 4. Discussion

Root-canal-treated teeth need to remain micro-organism-free, as an infection or reinfection would end up hindering the progress of the treatment. Inter-appointment intracanal medication prevents recontamination and counteracts the effects of micro-organisms that have resisted the cleaning and shaping procedures [40,41,42]. Among them, a combination of antibiotics is the most used for regenerative endodontic therapy [43,44,45].

The literature has shown that antibiotic pastes may reduce human dentin microhardness in vitro. In a clinical situation, this would be disadvantageous for immature teeth, because it could weaken the tooth, leading to fracture.

This study showed that, as expected, dentin microhardness decreased after having remained 20 days in contact with antibiotic medications pastes (TAP and DAP), as well as when in contact with Ca(OH)_2_. Contrary to our assumption, Ca(OH)_2_ affected dentin microhardness to a lesser extent in comparison with antibiotic pastes (with or without minocycline), regardless of the dentin location (closer or further from the canal lumen).

The most used intracanal medication in regenerative procedures is TAP [46,47], but DAP has also been used to avoid the tooth discoloration effect of minocycline [48]. Calcium hydroxide is an alternative used to disinfect the canal during endodontic regeneration [18,19].

Regenerative procedures are indicated to treat immature teeth with infected necrotic pulps, and the technique yields no or minimal mechanical shaping in the canal walls. This aims to prevent the removal of the dentinal structure because the tooth is already fragile [49,50]. To obtain the appropriate disinfection without shaping the canal walls, it is recommended that the intracanal medication with antibiotic pastes remain for approximately 21 days [13,51]. The medication concomitantly disinfects the environment and induces dentin apposition and root development [19]. The apposition of dentin and mineralized tissues would ultimately reduce the risk of fracture associated with traditional treatments, such as the apexification with Ca(OH)_2_ [47,52,53] or the MTA-apical plug technique [54,55].

Extrapolating the results obtained in this current study, the clinical use of antibiotic intracanal medication for 20 days would reduce dentin microhardness, which could in-duce the formation of a weakened intracanal structure. The dentin samples treated with TAP had an average reduction of approximately 24 KHN in relation to the baseline (dentin before treatment) (see Table 1). Similarly, DAP had an average reduction of 20 KHN. These values may not be clinically relevant; however, what we know for sure is that anti-biotic pastes reduced dentin microhardness more than Ca(OH)_2_—a material that has well-known negative effects on dentin [31,34]. This study showed that 20 days of medication with Ca(OH)_2_ promoted a reduction in dentin microhardness of only 10 KHN (on average).

The reduction in microhardness may have originated from the demineralizing effects of these acidic antibiotic mixtures when used at higher concentrations [34,56] (as with the ones used in this current study). The minocycline, present in TAP, is considered to be responsible for causing dentinal demineralization [31]. However, this current study did not find differences in microhardness reduction after treating dentin samples with an antibiotic paste with minocycline (TAP) or without minocycline (DAP).

The acidic characteristic of ciprofloxacin, metronidazole, and minocycline has the potential to demineralize the inorganic component of dentin. The monoclinic compound present in the crystal phase of pastes becomes attached to the calcium ions by chelation, inducing, therefore, a decrease in the hydroxyapatite of dentin [30,57,58]. A similar chelation process was demonstrated when root repair materials were exposed to acidic environments [59,60]. Chelating agents are substances that remove calcium ions from dentin, causing a decrease in microhardness and increasing dentin permeability [60]. Dentin erosion (the loss of calcium and phosphorous) is also reported upon the use of chelating substances, such as EDTA and TAP [53,61,62].

Dentin microhardness might vary depending on the proximity with the canal lumen [54,55]. When the medication is inserted into the root canal [33], a higher variation in microhardness may occur in the pre-dentin layer, i.e., closer to the canal lumen [63]. On the other hand, in experiments in which dentin samples are covered or immersed in the medication (as in this study), the same effect would occur regardless of the proximity to the canal lumen. This current study was accurate in showing the same reduction in dentin microhardness in different locations at the dentin wall (25 µm, 50 µm, or 100 µm). It is possible to understand that there was no variation depending on the proximity of the canal lumen; that is, regardless of the depth of penetration of the intracanal medication, the entire dentin wall will undergo changes in its microhardness, being susceptible to fracture, and these results differ from the results that were found by Haapasalo et al. (2014) [62] and Wang et. al. (2016) [64].

This study had strengths and weaknesses. It was valuable to show the amount of microhardness reduction in dentin samples treated with antibiotic pastes in comparison to the dentin treated with Ca(OH)_2_, which is a medication well-discussed in the literature. Also, this experiment did not use EDTA to wash the dentin samples before the immersion in the medications. It is known that EDTA irrigation (before or after the insertion of antibiotic pastes into the root canal) might result in a significant reduction in root dentin hardness ranging from 58% to 70% [30]. We had operators experienced and trained with the equipment and the methodology, which ensured the optimal standardization of the study protocol. A negative point of this study is the impossibility to say that the same results would occur in clinical practice [63] (and the challenges of a clinical study in testing identical outcomes). Another limiting factor is the difficulty (or even the impossibility) to obtain and standardize immature teeth. Furthermore, we (dentists) cannot control or predict the characteristics/mechanical properties (such as dentin composition, the arrangement of hydroxyapatite crystals, etc.) of a non-vital tooth in a clinical situation because they vary from patient to patient. These characteristics/mechanical properties are also altered and more unpredictable in non-vital teeth compared to vital teeth [65,66,67]. This ends up being a limitation of this study, since controlling these characteristics is a challenge for in vitro studies. Another possible limitation of the present study was the microhardness readings in different thirds of the dentin. The differences between the thirds of the root dentin were diluted in the same average per tooth. However, the study used 16 teeth per group, a highly robust number to bring reliability to the result. This high number of teeth per group enhanced the statistical analysis and diluted the variability in dentin morphology.

## 5. Conclusions

Based on the results of this in vitro study, the following conclusions were drawn:

- Ca (OH)_2_ is more appropriate to be used as intracanal medication for 20 days than antibiotic pastes (TAP or DAP) during regenerative procedures.
- Ca (OH)_2_ reduced dentin microhardness significantly less compared to the reduction caused by the antibiotic pastes (TAP or DAP), which have chelating properties.

Although microhardness has been used as an indicator of the overall resistance of dentin, it is important to highlight that the quality of dentin also depends on other properties, such as tensile strength, modulus of elasticity, etc.

## Figures and Tables

**Table 1:** Intra-group comparison in the same distances of dentin microhardness (KHN) at Day-0/baseline before immersion in the intracanal medications pastes: TAP (ciprofloxacin, metronidazole, and minocycline); DAP (ciprofloxacin and metronidazole); and Ca(OH)_2_ (calcium hydroxide paste, Ultracal^TM^ XS) (n = 16 teeth in each group, that originated 64 samples and 768 reading [12 reading in each sample]).

	Baseline without Medication (Day-0) Dentin Microhardness		
	25 µm	50 µm	100 µm
**TAP**	51.6 ± 23.4 ^A^	50.5 ± 16.7 ^A^	52.7 ± 19.9 ^A^
**DAP**	48.8 ± 16.6 ^A^	48.9 ± 15.5 ^A^	49.6 ± 16.0 ^A^
**Ca(OH)_2_**	55.1 ± 24.6 ^A^	50.8 ± 17.8 ^A^	55.7 ± 19.1 ^A^

^A^ Different uppercase letters in the same column indicate significant difference between groups, in the same distance [Mann–Whitney’s and Kruskal–Wallis’ tests (α = 5%)].

**Table 2:** Intra-group comparison in the same distances of dentin microhardness (KHN) at Day-20 after immersion in the intracanal medications pastes: TAP (ciprofloxacin, metronidazole, and minocycline); DAP (ciprofloxacin and metronidazole); and Ca(OH)_2_ (calcium hydroxide paste, Ultracal^TM^ XS) (n = 16 teeth in each group, that originated 64 samples and 768 reading [12 reading in each sample]).

	After-Medication (Day-20) Dentin Microhardness		
	25 µm	50 µm	100 µm
**TAP**	28.0 ± 4.5 ^A^	29.5 ± 9.1 ^A^	28.9 ± 9.5 ^A^
**DAP**	29.8 ± 7.7 ^A^	30.2 ± 6.1 ^A^	29.6 ± 5.5 ^A^
**Ca(OH)_2_**	44.2 ± 24.4 ^B^	41.7 ± 24.5 ^B^	39.9 ± 18.8 ^B^

^A/B^ Different uppercase letters in the same column indicate significant difference between groups, in the same distance [Mann–Whitney’s and Kruskal–Wallis’ tests (α = 5%)].

## Data Availability

The data are available from the study authors and can be requested, as they are not available via a link.
